# Factors influencing low-income households’ food insecurity in Bangladesh during the COVID-19 lockdown

**DOI:** 10.1371/journal.pone.0267488

**Published:** 2022-05-10

**Authors:** Suvasish Das Shuvo, Md. Sakhawot Hossain, Md. Riazuddin, Sanaullah Mazumdar, Deepa Roy

**Affiliations:** 1 Department of Nutrition and Food Technology, Jashore University of Science and Technology, Jashore, Bangladesh; 2 Department of Mathematics, Jashore University of Science and Technology, Jashore, Bangladesh; The Technical University of Kenya, KENYA

## Abstract

**Background:**

The COVID-19 pandemic and countrywide lockdown could negatively impact household food insecurity among low-income households. This study aimed to investigate the prevalence of household food insecurity and its influencing factors among low-income people in Bangladesh during the lockdown of COVID-19.

**Methods:**

This cross-sectional study was conducted through face-to-face interviews from 500 low-income households during the countrywide COVID-19 lockdown. A pretested, structured and validated questionnaire was used to collect socioeconomic characteristics, household income conditions, and food accessibility. Household Food Insecurity Access Scale (HFIAS) and Dietary Diversity Score (DDS) were used to measure food insecurity. Multinomial logistic regression models were estimated to evaluate and predict risk factors that influence food insecurity.

**Results:**

The study found that above 67% of households was mild-to-moderate food insecure while 23% experienced severe food insecurity. Significantly, 88%, 97.4%, and 93.4% of the households had anxiety and uncertainty, inadequate quality, and inadequate quantity of food, respectively. The regression analysis revealed the age 36–50 years (RRR: 4.86; 95% CI: 2.31–7.44, RRR: 4.16; 95% CI: 2.25–6.10) and monthly income <58.3 USD (RRR: 3.04; 95% CI: 1.12–5.14, RRR: 3.26; 95% CI: 1.79–4.71) were significantly associated with food insecurity (p <0.001). Likewise, less-income (RRR: 3.87; 95% CI: 1.37–6.46, RRR: 2.99; 95% CI: 1.16–4.83), increase in food prices (RRR: 1.29; 95% CI: 0.32–2.33, RRR: 1.08; 95% CI: 0.05–1.12), and those who did not have same type of earning as before during the COVID-19 lockdown (RRR: 3.41; 95% CI: 1.33–5.62, RRR: 2.60; 95% CI: 0.99–4.24) were potential risk factor for MMFI and FI.

**Conclusion:**

This study found that households become more susceptible to food insecurity during the COVID-19 pandemic and lockdown period. Based on the findings, we suggest some essential food policies and adequate food assistance to mitigate these negative consequences.

## 1. Introduction

A newly discovered coronavirus (COVID-19) pandemic poses a grave public health threat to development around the world [1]. Though this pandemic directly hit human health but indirectly led to a catastrophic challenge to food insecurity (FI) through disruptions in health and nutrition services, food supply chains, and livelihoods [2, 3]. Additionally, low-income households are disrupted by the pandemic in several ways comprising unemployment and low wages, movement restrictions, and household stress [1, 4]. According to the United Nations World Food Programme, above 820 million people in lower and middle-income countries went to sleep hungry where 135 million people had already experienced acute food insecure even earlier than the COVID-19 pandemic [5]. In this circumstance, in lower-middle-income countries like Bangladesh, approximately 51 million people were moderately or severely food insecure before the COVID-19 [6]. Moreover, the Bangladesh Institute of Development Studies reported that 25.5 million new people are expected to join the extreme poverty club and 13% of people lost their works due to the pandemic lockdown [7]. Besides, recent studies in Bangladesh reported that about 90% of the households were experiencing different grades of food insecurity during the strict lockdown period [8, 9].

As the number of COVID-19 infections rises worldwide, many countries like Bangladesh decided to impose a countrywide lockdown to reduce the spread of the coronavirus [8]. From April 14, 2021, the Bangladesh government imposed a strict lockdown across the country to battle the second wave of the COVID-19 epidemic [10]. As the infection rate was gradually increased, the Government was forced to extend the ongoing strict lockdown to slow down the rapid spread of COVID-19 infection. The government effectively implemented the lockdown by imposing strict travel restrictions, stay-at-home orders, and market/shopping mall closures, leading to a sudden drop in food security among low-income people residents [11]. Adverse effects on domestic food supply chains, shocks in food production, and income losses are causing mental stress and food insecurity during the strict lockdown. The majority of the low-income earners, daily laborers, and workers from different informal sectors reported zero income during the entire period of lockdown [11, 12]. Households are found to cut down the quantity and quality of food consumption because of the higher retail price and reduced income [1, 9]. A recent countrywide study by BRAC showed that 93% of the low-income respondents had faced a loss of earnings, where 54% of households had no income, and 14% of households had no reserve food [13].

Food insecurity is defined as the limited or uncertain access to sufficient nutritious food for an active and healthy life. The four components that widely affect food insecurity are *availability*, *accessibility*, *utilization*, and *stability* [6, 14]. Food insecurity is also categorized by mild-to-moderate food insecurity (MMFI), and severe food insecurity (SFI). These two forms of food insecurity also hit the poor households by three domains including anxiety and uncertainty about the household food supply, inadequate quality, and insufficient quantity of food intake [6, 14]. The unprecedented Covid-19 pandemic, and the associated social and economic response-related factors like movement restrictions, income losses, poor income, lack of access to adequate food, and increasing price have led to proliferate food insecurity and its related health disparities among the at-risk populations [9, 15]. Some other factors that indirectly influence food insecurity are occupation, monthly household income, education, family size, and others [16]. Importantly, these factors also boost as another supporting indicator of measuring FI is dietary diversity score (DDS) [17].

Several studies have evaluated the impact of the Covid-19 lockdown (or quarantine) on food insecurity in different countries [4, 8, 18–24]. Some systematic studies reported that the COVID-19 pandemic inversely affected food access, supply, demands, and decreased purchasing power resulting in to increase in the prevalence of household food insecurity [8, 9, 23, 25]. However, the prevalence of food security and its associated factors among low-income people in Bangladesh still remain to unfold. Furthermore, there is an absence of empirical evidence of the changes in types and quantity of food diversity in low-income workers during the pandemic. Considering the above facts, it’s very indispensable to investigate the relation between the COVID-19 pandemic and household food insecurity among low-income people in Bangladesh. This study will offer evidence on the association between the COVID-19 pandemic and household food insecurity. The outcome of the present study will assist the government authorities and policymakers through identifying the associated factors that affect household food insecurity among low-income people in Bangladesh as well as in developing effective appropriate policies and strategies for combating the issue of food security as fallout of Covid-19. With this multifold viewpoint, this study aims to explore the prevalence of household food insecurity and its influencing factors among low-income people in Bangladesh during the COVID-19 lockdown period.

## 2. Methods

### 2.1 Study design and setting

**Fig 1:**
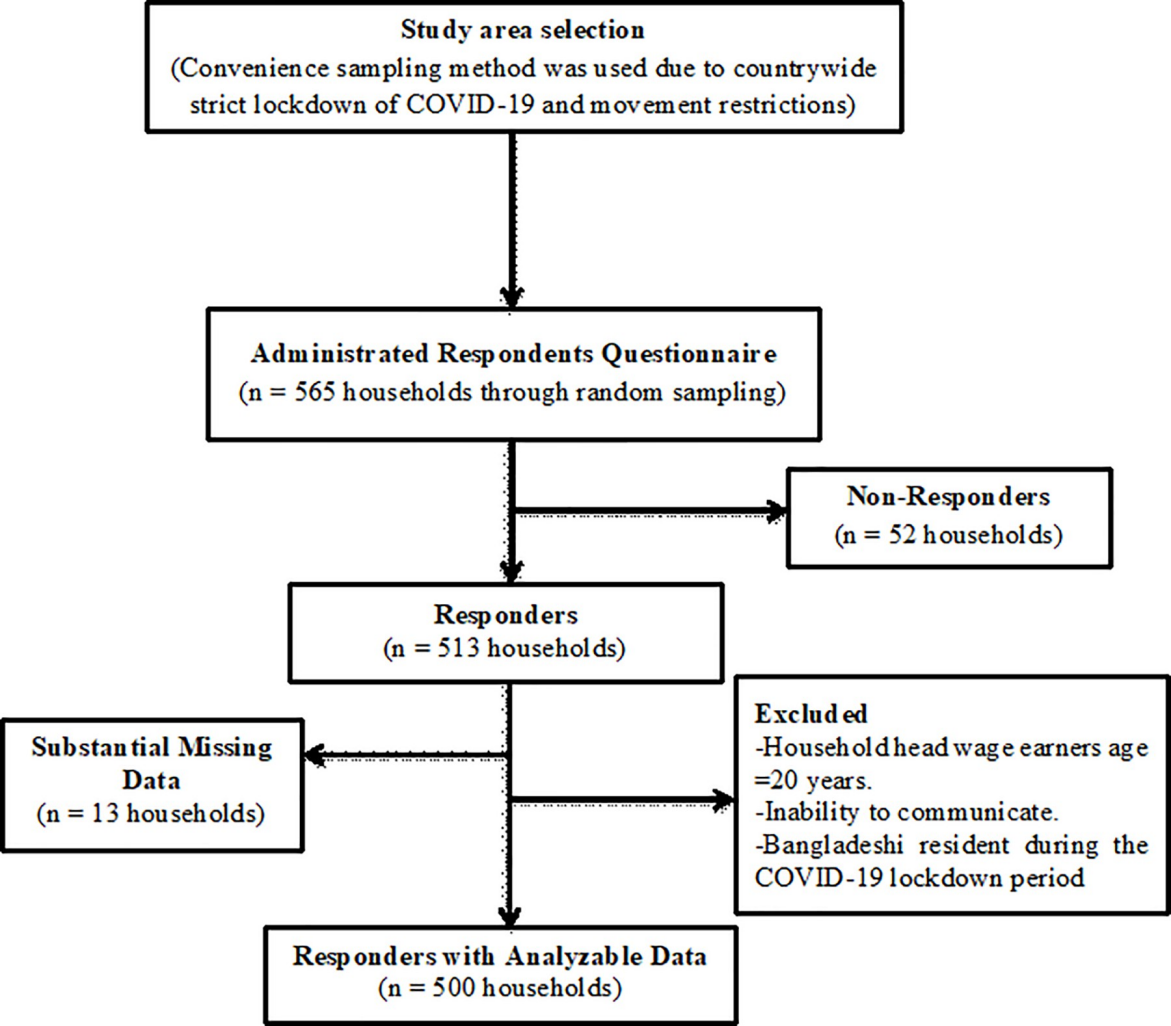
Flow chart of respondent’s recruitment.

A cross-sectional study was conducted to evaluate the prevalence of household food insecurity and associated factors during the COVID-19 lockdown among Bangladeshi low-income people from 3 May to 15 May 2021. We estimated sample size based on an unknown prevalence of household food security (therefore considering 50% prevalence) with a 5% margin of error to be tolerated at the 95% level of confidence, and 95% response rate. On this basis, a total of 500 household heads >20 years old and above agreed to participate in the study. In this study, three divisions of Bangladesh (Rajshahi, Chattogram, and Khulna) were selected using convenience sampling methods because of movement restrictions caused by the countrywide strict lockdown. Then five urban areas from each division were randomly included for data collection. Respondents who worked as daily wage workers to support their families were recruited using a simple random sampling technique. The main inclusion criteria of the surveyed study were household head wage earners aged>20 years, inability to communicate, and Bangladeshi residents during the COVID-19 lockdown period (Fig 1).

### 2.2 Data collection procedures

A structured validated questionnaire was used to collect the data, which consisted of four sections including socio-demographic characteristics, the frequency of food accessibility, household food insecurity, and dietary diversity score during the lockdown period of the COVID-19 pandemic. A face-to-face interview was conducted by the four trained interviewers by maintaining social distance and proper safety measures (mask, gloves, and hand sanitizer) during the strict lockdown in Bangladesh. The interviewers who were selected have educational backgrounds in nutrition and food technology and previous experience in administering health surveys. Interviewers also received training on study tools, participant recruitment, ethical considerations, and data collection techniques. Respondents were given enough time to write down their responses to each question. The questionnaire was written in English at first, and then the questions were translated into the respondents’ native language (Bengali) to make them more understandable [26]. The enumerator informed the respondents prior to data collection and assured them that the provided information would be kept confidential. To ensure clarity and eliminate any irrelevant and repeated questions, the questionnaire was pilot-tested with small samples (40 households) in face-to-face surveys before the final survey.

### 2.3 Socio-economic characteristics and food accessibility

The socio-economic variables comprised of seven questions including age, gender, level of education, occupation, monthly income, family members, and marital status. The food accessibility section included nine items regarding food accessibility during COVID-19 lockdown such as effect on income, change in the type of food cooked, change in cooking frequency, reasons for the change in the type of food cooked, food sources, increases of food prices, get the same amount of food as before, get the same type of food as before, and get the same type of income as before [8, 27].

### 2.4 Household food insecurity access

The FAO-FANTA guideline’s Household Food Insecurity Access Scale (HFIAS) was used to assess household food insecurity access [28]. The HFIAS questionnaire consists of nine occurrence questions that present the increasing level of severity of food insecurity in the last month. Each occurrence of the question consisted of two response options including non-occurrence (no were coded as 0) and occurrence (yes were coded as 1). Besides the HFIAS frequency-of-occurrence questions were also asked each respondent as a follow-up to each occurrence question to assess how often the condition occurred. Each frequency of occurrence question comprised of three responses was assessed using a three-point scale non-occurrence, rarely (once or twice in the past four weeks), sometimes (three to ten times in the past four weeks), and often (more than ten times in the past month) ranging from 0 to 3, respectively. For nine items based on the frequency of occurrence, the possible score ranges from 0–27, whereas the higher score represents the more food insecure and the lower score represents the more food secure household. Then, household food insecurity (access) was reported through the indicator of Household Food Insecurity Access Prevalence (HFIAP) Status. The HFIAP indicator is categorized into three levels of household food insecurity (access) including food security, mild-to-moderate food insecurity, and severe food insecurity. As households answered affirmatively to more severe conditions and/or experienced those conditions more frequently, they were classified as becoming increasingly food insecure. HFIAS indicator guide was used to measure the household food insecurity status [8, 28].

### 2.5 24-hours household dietary diversity score

The respondents’ Household Dietary Diversity Scores (DDS) were calculated using data from the 24-hour dietary recall developed by FAO and the FANTA Project [29]. Twelve food groups were included in DDS. A single point was awarded to each of the food groups consumed during the reference period, yielding a maximum total dietary diversity score of 12 points for each individual if his/her responses were positive to all food groups. The total dietary diversity score (DDS) ranges from 0 to 12. The DDS scores were categorized into three categories including low dietary diversity score (0–3), moderate dietary diversity score (4–6), and high dietary diversity score (7–12) [29, 30].

### 2.6 Statistical analysis

Firstly, this study described the overall socioeconomic characteristics, food accessibility, household food insecurity, and dietary diversity score using frequency, and percentages by Chi-square test. Secondly, the potential uncertainty and bias of univariate analysis have emphasized the need for *multinomial logit model* analysis that the prevalence of household food insecurity and associated factors during the COVID-19 lockdown. In contrast age, level of education, occupation, monthly income, family members, marital status, household dietary diversity score (HDDS), effect on income conditions, and food accessibility during COVID-19 lockdown data are discrete data. The database used in this study comprises three discrete categories for the prevalence of household food insecurity including food secure, mild-to-moderate food insecure, and severely food insecure. The classification of household food insecurity access prevalence status is based solely on the individual household food security status. In this case, an ordered probability model might be the most feasible. A multinomial logit model for food security levels was employed to explain the severity of food insecurity and its associated factors among the three categories of food security status, following the usual use of unordered discrete outcome models. This model permits the use of a categorical dependent variable [31].

The probability of the severity of food insecurity and its associated determinants during COVID-19 lockdown can be stated as follows in the general case of a random effect context of the severity of food insecurity outcomes:

$$Y_{ki}=\alpha_{k}+\beta_{k}X_{ki}+\epsilon_{ki}$$ (1)

Where, *Y*_*ki*_ is an outcome variable such as the different types of food security levels *k* (*k* = 1,2,3) of sample *i* (*i* = 1,……*n*, where *n* is the total number of observations), *α*_*k*_ is a constant parameter for category *k*; *β*_*k*_ is a vector of the estimable parameters of each category; *X*_*ki*_ represents a list of explanatory variables that are responsible for determining the intensity of food insecurity outcomes and *ϵ*_*ki*_ is a random error term that is independently and identically distributed. As the samples are falling into different types of food security levels, therefore, with the assumption that the error term (*ϵ*_*ki*_) following the generalized extreme value (i.e., Gumbel) distribution, the probability of falling food insecurity types *k* of sample *i*, conditioning on *X*_*ki*_ and *α*_*k*_. Hence, the *multinomial logit model* forms as follows:

$$P_{k}=Pr[Y_{i}=k]==\frac{\exp\left(\alpha_{k}+\beta_{k}X_{ki}\right)}{\sum_{⩝k}\exp\left(\alpha_{k}+\beta_{k}X_{ki}\right)}$$ (2)

In our empirical framework, the estimated coefficients were used to evaluate the probabilities of food insecurity falling into one of the three categories. Our model consists of three probabilities, *P*_*k*_ (*k = 1*,*2*,*3*), related to the three categories of food insecurity (i.e. food secure, mild-to-moderate food insecure, and severely food insecure). The probability of being food secure, mild-to-moderate food insecure, and severely food insecure are denoted as *P*_*1*_, *P*_*2*_, and *P*_*3*_ respectively. Because the probabilities of falling different types of food security levels are conditioned on *α*_*k*_. As a result, the sample likelihood of the multinomial logit model (Eq 2) follows the standard maximum likelihood method. Since the explanatory variables are continuous and discrete determining factors log-odd ratios of the outcomes become:

$$\ln(\frac{\mathrm{Pr}\left(Y_{i}=k-1\right)}{\mathrm{Pr}\left(Y_{i}=k\right)})=\beta_{i}X_{k}-\beta_{n}X_{k}=(\beta_{i}-\beta_{n})X_{k}$$ (3)

Thus, the coefficients are distinguishable only up to an additive constant so only the difference in coefficients is identifiable. One outcome (the base category) of the coefficient is fixed zero to resolve this inter determinacy. Due to the non-linear characteristics of the *multinomial logit model*, the estimated coefficients of the independent variables do not represent their effects on the dependent variable. In that manner, the relative risk ratio (RRR) represents the effect of a relevant risk factor. In our analysis, the RRR of risk factors is computed relative to the base category (i.e. food secure). For instance, the relative probability of food insecurity outcome (*k* = 2) to the base category (*k* = 1) is given as:

$$\frac{Pr(k=2)}{Pr(k=1)}=\exp\left({x\beta }^{\left(k=2\right)}\right)$$ (4)

Therefore, the RRR is written as

$$RRR=\exp\left({x\beta }^{\left(k=2\right)}\right)$$ (5)

Eqs (4) and (5) imply the RRR of mild-to-moderate food insecure (*k* = 2) relative to the food secure (*k* = 1) category. Similarly, if we consider *k* = 3 t will indicate the RRR of severely food insecure relative to food secure (*k* = 1) types. Moreover, the intuition of RRR of an independent variable indicates the increase (RRR > 1) or decrease (RRR< 1). In this study, the *multinomial logit model* and the associated RRR were estimated using Stata (version 14.0). All associated factors of food insecurity during the COVID-19 lockdown were entered into the same model. The model was adjusted for potential confounding by age, level of education, occupation, monthly income, family members, marital status, HDDS, and effect on income conditions and food accessibility during COVID-19 lockdown, which has been influenced the risk of household food insecurity. The Hosmer and Lemeshow goodness-of-fit test was used to determine the final model, and the Wald test was used to evaluate the significance of variables. The statistical significance test was two-sided and a p-value <0.05 was considered for fitting the multinomial logistic regression models.

### 2.7 Ethical approval

The study protocol was approved by the institutional review board of the Faculty of Biological Science and Technology, Jashore University of Science and Technology, Bangladesh (Ref: ERC/FBST/JUST/2021-57). Both written and oral informed consent was sought from the participants (thumb impressions from those who were not able to read and write) before administering the survey.

## 3. Results

### 3.1 Socio-economic characteristics of the study sample

A total of 500 low-income households participated in this study, where 42.8% of respondents were aged between 21 and 35 years and 38.2% were aged between 36 and 50 years (Table 1). A majority of the respondents were completed primary education (23.6%) and secondary education (45%). Nearly 37% of the household heads were day laborers, and most of the households had a low income of 58.4–116.6 USD per month (50.8%). As such, around 45% of households consist of 4–5 persons. More than three-fourths of the respondents were married (89%). In addition, around two-thirds of the respondents (65.6%) had a low DDS, whereas only 13.4% had high DDS.

**Table 1:** Characteristics of respondents based on household food insecurity access proportion (n = 500).

Variables	Categories	Total	FS	MMFI	SFI	P-value
		n (%)	n (%)	n (%)	n (%)	
**Age**	21–35 years	214 (42.8)	20 (9.4)	156 (72.9)	38 (17.7)	0.176
	36–50 years	191 (38.2)	18 (9.4)	117 (61.3)	56 (29.3)	
	51–65 years	86 (17.2)	9 (10.4)	57 (66.3)	20 (23.3)	
	>65 years	9 (1.8)	0 (00)	7 (77.8)	2 (22.2)	
**Level of education**	Illiterate	97 (19.4)	9 (9.3)	66 (68.0)	22 (22.7)	0.116
	Primary	118 (23.6)	17 (14.4)	81 (68.6)	20 (17.0)	
	Secondary	225 (45.0)	14 (6.2)	155 (68.9)	56 (24.9)	
	Higher Secondary	60 (12.0)	7 (11.7)	35 (58.3)	18 (30.0)	
**Occupation**	Day laborer	183 (36.6)	13 (7.1)	134 (73.2)	36 (19.7)	**<0.001**
	Rickshaw puller	120 (24.0)	6 (5.0)	83 (69.2)	31 (25.8)	
	Hotel Worker	141 (28.2)	12 (8.5)	93 (65.9)	36 (25.6)	
	Others	56 (11.2)	16 (28.6)	27 (48.2)	13 (23.2)	
**Family income per month**	<58.3 USD	178 (35.6)	6 (3.4)	107 (60.1)	65 (36.5)	**<0.001**
	58.4–116.6 USD	254 (50.8)	15 (5.9)	196 (77.2)	43 (16.9)	
	116.7–174.9 USD	51 (10.2)	15 (29.4)	29 (56.9)	7 (13.7)	
	>175 USD	17 (3.4)	11 (64.7)	5 (29.5)	1 (5.8)	
**Family member**	2–3	65 (13.0)	10 (15.4)	40 (61.5)	15 (23.1)	0.265
	4–5	223 (44.6)	15 (6.7)	163 (73.1)	45 (20.2)	
	6–7	150 (30.0)	18 (12.0)	94 (62.7)	38 (25.3)	
	≥8	62 (12.4)	4 (5.9)	40 (64.7)	18 (29.4)	
**Marital Status**	Unmarried	35 (7.0)	5 (14.3)	27 (77.1)	3 (8.6)	**0.05**
	Married	445 (89.0)	38 (8.5)	296 (66.6)	111 (24.9)	
	Widowed	20 (4.0)	4 (20.0)	14 (70.0)	2 (10.0)	
**Dietary Diversity Score (DDS)**	High DDS	67 (13.4)	45 (67.1)	21 (31.3)	1 (1.6)	**0.002**
	Moderate DDS	105 (21.0)	31 (29.6)	53 (50.5)	21 (19.9)	
	Low DDS	328 (65.6)	11 (3.6)	231 (70.1)	86 (26.3)	

**Note:** FS: food security; MMFI: mild-to-moderate food insecurity; SFI: severe food insecurity; DDS: dietary diversity score.

### 3.2 The proportion of food insecurity status in the study area during the lockdown period

The proportion of Household Food Insecurity status among low-income people is presented in Table 3. The survey result shows that above 67% and 23% of households had mild-to-moderate food insecurity (MMFI) and severe food insecurity (SFI) whereas only 9.4% of households had food security (FS). Almost all of the socioeconomic characteristics dispersed more or less the same portion within food insecurity including MMFI, and SFI as shown in Table 1. The findings revealed that about 43% and 38.2% of the household heads aged between 21–35 years and 36–50 years were food insecure, respectively (72.9% and 61.3% MMFI, 17.7%, and 29.3% SFI). In terms of education, 45% of the secondary educated respondents were MMFI (68.9%) and SFI (24.9%). Whereas 36.6% and 28.2% of the household heads who were day laborers and rickshaw pullers were MMFI (73.2% and 69.2%), and SFI (19.7% and 25.8%) respectively. 89% of households heads who had married were food insecure (66.6% MMFI and 24.9% SFI). Remarkably, above two-thirds of the total respondents with very DDS had MMFI (70.1%) and 26.3% had SFI.

Table 2 displays the proportion of income conditions and food accessibility during COVID-19 among respondents with household food insecurity. Most of the household heads claimed that daily income was affected due to the COVID-19 lockdown period, whereas 42.2% had less income (not enough for food) and also suffered from food insecurity (68.7% mild to moderate food insecure). Above 75% of respondents who had changed their type of food cooked during COVID-19 lockdown were food insecure (67% MMFI and 26.1% SFI). Lower-income (67%) and more people in the households (20.8%) were the most frequently mentioned reasons for the change in the type of food cooked than usual during the lockdown. Local shop or market (76.4%) was the main food source during COVID-19 lockdown, whereas only a small portion of households (13%) got government relief or different help assistant. Almost all of the respondents (95%) reported that food prices were increased during COVID-19 lockdown and they were also experienced to MMFI (68%), and SFI (22.9%). At the time of COVID-19 lockdown, most of the respondents who did not get the same type of income (92.4%), the same amount of food (87.2%), and the same type of food (89.8%) as before were food insecure (70.4%, 72.5%, and 69.1% MMFI; 23.8%, 24.1%, and 24% SFI).

**Table 2:** Effect on income conditions and food accessibility during COVID-19 among respondents with household food insecurity (n = 500).

Variables		Total		FS	MMFI		SFI		P-value
		n (%)		n (%)	n (%)		n (%)		
**Effect on income during COVID-19 lockdown**									
No change	69 (13.8)		14 (20.3)		44 (63.8)		11 (15.9)		**0.007**
Less income (not enough for food)	211 (42.2)		4 (8.1)		145 (68.7)		49 (23.2)		
Less income (but enough for food)	176 (35.2)		14 (7.9)		123 (69.9)		39 (22.2)		
No income coming into household	44 (8.8)		2 (4.5)		25 (56.8)		17 (38.7)		
**Change in type of food cooked during COVID-19 lockdown**									
No	124 (24.8)		21 (16.9)		85 (68.6)		18 (14.5)		**0.003**
Yes	376 (75.2)		26 (6.9)		252 (67.0)		98 (26.1)		
**Change in cooking frequency during COVID-19 lockdown**									
No change		98 (19.6)	15 (15.3)		67 (68.4)		16 (16.3)		**0.002**
More frequent		57 (11.4)	0 (00)		33 (57.9)		24 (42.1)		
Much less frequent		74 (14.8)	7 (9.5)		50 (67.6)		17 (22.9)		
Less frequent		271 (54.2)	25 (9.3)		187 (69.0)		59 (21.7)		
**Reasons for change in the type of food cooked during COVID-19 lockdown**									
More people in household		104 (20.8)		9 (8.6)		76 (73.2)	19 (18.3)		0.209
Lower availability of cooking fuel		6 (1.2)		0 (00)		4 (66.7)	2 (33.3)		
Lower availability of food		55 (11.0)		10 (18.2)		34 (61.8)	11 (20.0)		
Lower income		335 (67.0)		28(8.3)		223 (66.6)	86 (25.1)		
**Food source during COVID-19 lockdown**									
Local shop/market (same as before lockdown)		382 (76.4)		39 (10.2)		253 (66.2)		90 (23.6)	0.855
Local shop/market (different location than before lockdown)		39 (7.8)		2(5.2)		30 (76.9)		7 (17.9)	
Source from Govt. Relief /Help assistant		65 (13.0)		5 (7.7)		45 (69.2)		15 (23.1)	
Friends/family/source from home (different than before lockdown)		14 (2.8)		1 (7.1)		9 (64.3)		4 (28.6)	
**Increase of food prices due to COVID-19 lockdown**									
No		12 (2.4)		3 (25.0)	5 (41.7)			4 (33.3)	0.291
Don’t know		13 (2.6)		1 (7.7)	9 (69.2)			3 (23.1)	
Yes		475 (95.0)		43 (9.1)	333 (68.0)			109 (22.9)	
**Get the same amount of food as before the COVID-19 lockdown**									
No	436 (87.2)		15 (3.4)		316 (72.5)			105 (24.1)	**<0.001**
Yes	64 (12.8)		32 (50.0)		21 (32.8)			11 (17.2)	
**Get the same type of food as before the COVID-19 lockdown**									
No	449 (89.8)		31 (6.9)		310 (69.1)		108 (24.0)		**<0.001**
Yes	51 (10.2)		16 (31.4)		27 (52.9)		8 (15.7)		
**Get the same type of income as before the COVID-19 lockdown**									
No	462 (92.4)		27 (5.8)		325 (70.4)		110 (23.8)		**<0.001**
Yes	38 (7.6)		20 (52.6)		12 (31.6)		6 (15.8)		

**Note:** FS: food security; MMFI: mild-to-moderate food insecurity; SFI: severe food insecurity.

Table 3 presents the summary information on the proportion of households experiencing one or more behaviors in each of the three domains reflected in the HFIAS—Anxiety and uncertainty, Insufficient quality, and Insufficient quantity of food intake and its physical consequences. In total, 88% of households had experienced anxiety about their food supply. Surprisingly, more than 97% and 93% of households had an inadequate quality of food and insufficient food intake during the COVID-19 lockdown period respectively.

**Table 3:** Frequency of occurrence of nine conditions of Household Food Insecurity Access Scale (HFIAS) and proportion of household food insecurity status during the COVID-19 lockdown.

**(i) Frequency of occurrence of nine conditions of Household Food Insecurity Access Scale (HFIAS) (n, (%))**					
**Domain**	**HFAIS Conditions**	**Never**	**Rarely**	**Sometimes**	**Often**
		**n (%)**	**n (%)**	**n (%)**	**n (%)**
**Anxiety and uncertainty about the household food supply**	(Q1) Worry about food	60 (12.0)	78 (15.6)	299 (59.8)	63 (12.6)
**Inadequate quality of food**	(Q2) Unable to eat preferred foods	42 (8.4)	90 (18.0)	221 (44.2)	147 (29.4)
	(Q3) Eat a limited variety of foods	32 (6.4)	126 (25.2)	235 (47.0)	107 (21.4)
	(Q4) Eat foods that you did not want to eat	47 (9.4)	75 (15.0)	165 (33.0)	213 (42.6)
**Insufficient food intake**	(Q5) Eat a smaller meal	41 (8.2)	109 (21.8)	287 (57.4)	63 (12.6)
	(Q6) Eat fewer meals in a day	394 (78.8)	29 (5.8)	24 (4.8)	53 (10.6)
	(Q7) No food to eat of any kind in the household	472 (94.4)	15 (3.0)	5 (1.0)	8 (1.6)
	(Q8) Go to sleep at night hungry	458 (91.6)	22 (4.4)	10 (2.0)	10 (2.0)
	(Q9) Go a whole day and night without eating	486 (97.2)	7 (1.4)	1 (0.2)	6 (1.2)
**(ii) Household Food Insecurity Access-related Domains (Yes to at least one condition of a domain (n, (%))**					**Total n (%)**
Anxiety and uncertainty about the household food supply					440 (88)
Inadequate quality of food					487 (97.4)
Insufficient food intake					467 (93.4)
**(iii) Proportion of Household Food Insecurity Status**					**Total n (%)**
Severely food insecure					47 (9.4)
Mild-to-moderate food insecure					337 (67.4)
Food secure					116 (23.2)

**Note:** HFIAS: Household Food Insecurity Access Scale

### 3.3 Factors associated with the households’ food insecurity among the respondents during the lockdown

The determinants of household food insecurity during the COVID-19 pandemic lockdown are presented as the relative risk in Table 4. Respondents aged between 36–50 years and 21–35 years were 4.86 (95% CI: 2.31–7.44) times and 3.87 (95% CI: 1.90–5.84) times more likely to be mild-to-moderately food insecure (MMFI) but aged 36–50 years were 4.16 times more likely to be severely food insecure (SFI) than above 65 years aged respondents. Respondents with secondary education and illiterate were 3.73 (95% CI: 1.72–6.13) times and 2.97 (95% CI: 1.19–4.87) times more likely to be MMFI than households whose heads had higher secondary education. Additionally, respondents who had secondary education and illiterate were 2.79 times and 2.39 times higher odds of being SFI than higher secondary respondents. In terms of occupation, this study found that households that rely on day labor jobs and rickshaw pullers were 3.10 (95% CI: 1.77–4.49) times and 4.54 (95% CI: 1.96–7.38) times more likely to be MMFI than households that rely on others occupations but among the respondents, the odds of being SFI were 3.96 times higher among rickshaw puller.

**Table 4:** Multivariate logit regression model results for the determinants of household food insecurity.

Variables	Mild-to-moderately food insecurity Versus Food security	Severe food insecurity Versus Food security
	*RRR (95%CI)*	*RRR (95%CI)*
**Age**		
**>65 years**	1	1
51–65 years	1.52 (0.42–2.60)	2.99 (0.76–5.25)
36–50 years	**4.86 (2.31–7.44)** ***	**4.16 (2.25–6.10)** ***
21–35 years	**3.87 (1.90–5.84)** **	**2.32 (0.84–3.77)** **
**Level of education**		
Higher secondary	1	1
Secondary	**3.73 (1.72–6.13)** **	**2.73 (1.30–3.86)** **
Primary	**2.19 (1.09–3.48)** **	**2.22 (1.13–3.61)** **
Illiterate	**2.97 (1.19–4.87)** ***	**2.39 (1.19–4.07)** ***
**Occupation**		
Others	1	1
Day laborer	**3.10 (1.77–4.49)** **	2.64 (1.15–4.17)
Rickshaw puller	**4.54 (1.96–7.38)** **	**3.96 (1.55–6.45)** **
Hotel Worker	2.55 (1.37–4.34)	1.88 (0.18–3.62)
**Family income per month**		
Above 175 USD	1	1
116.7–174.9 USD	1.78 (0.45–3.26)	1.44 (0.18–1.79)
58.4–116.6 USD	**4.59 (1.61–7.88)** **	**2.91 (0.96–4.86)** **
<58.3 USD	**3.04 (1.12–5.14)** ***	**3.26 (1.79–4.71)** ***
**Family member**		
2–3	1	1
4–5	**2.37 (0.67–4.23)** **	**2. 91 (1.52–4.48)** **
6–7	2.12 (0.78–3.53)	2. 17 (0.78–3.57)
≥8	**3.01 (1.23–5.11)** **	**1.35 (0.31–2.44)** **
**Marital Status**		
Unmarried	1	1
Married	**4.38 (1.33–7.61)** ***	**3.55 (2.38–4.69)** ***
Widowed	1.07 (0.38–1.79)	1.92 (0.82–3.17)
**DDS**		
High DDS	1	1
Moderate DDS	2.58 (1.14–4.38)	0.87 (0.13–1.68)
Low DDS	**4.92 (1.87–7.61)**	**2.49 (0.94–2.94)**
**Effect on income**		
No change	1	1
Less income (not enough for food)	**3.87 (1.37–6.46)** ***	**2.99 (1.16–4.83)** ***
Less income (but enough for food)	1.62 (0.13–2.26)	1.42 (0.47–2.38)
No income coming into household	2.67 (1.13–4.43)	1.67 (0.15–3.27)
**Change in type of cooked food**		
No	1	1
Yes	2.62 (1.36–3.78)	1.81 (0.20–3.43)
**Reasons for change in the type of cooked food**		
No reason	1	1
More people in household	**2.65 (1.12–4.13)** **	**2.32(0.84–2.87)** **
Lower availability of food	1.29 (0.55–2.12)**	1.52 (0.08–3.07)
Poor income	**3.43 (1.48–5.46)** ***	**2.70 (1.19–4.23)** ***
**Increase in food prices**		
No	1	1
Don’t know	0.99 (0.03–1.98)	0.20 (0.04–0.36)
Yes	1.29 (0.32–2.33)	1.08 (0.05–1.12)
**Get the same quantity of food as before**		
Yes	1	1
No	**3.21 (1.47–5.04)** **	**3.40 (1.50–5.41)** **
**Get the same quality of food as before**		
Yes	1	1
No	1.18 (0.45–1.96)	1.99 (0.49–3.50)
**Earned the same type of income as before**		
Yes	1	1
No	**3.41 (1.33–5.62)** ***	**2.60 (0.99–4.24)** ***
Observation	500	
Log-likelihood	-250.73	
*P*-value	<0.001	
LR chi^2^ (*χ*^*2*^)	200.65	
Pseudo R^2^ (*ρ*^*2*^)	0.27	

**Note:** FS: food security; MMFI: mild-to-moderate food insecurity; SFI: severe food insecurity; DDS: dietary diversity score. The dependent variable is the food security status (outcome: mild-to-moderate food insecurity and severe food insecurity; reference: food security). The model includes the age, occupation, and education of the household head, family income per month, household size, and marital status of household head, DDS, and household food access. Relative risk ratios are presented and 95% confidence intervals are in parenthesis.

*** p<0.01

** p<0.05

* p<0.1

The households with a monthly income of <58.3 USD were 3.04 times more likely to experience MMFI and 3.26 times more likely to experience SFI during the COVID-19 pandemic lockdown than households whose income was above 175 USD during the same period. Again, respondents with the monthly income of 58.4–116.6 USD and 116.7–174.9 USD were 4.59 times and 1.78 times more likely to experience MMFI, respectively. The number of family members was significantly associated with MMFI and SFI. The households that had 4–5 family members and ≥8 family members were 2.37 (95% CI: 0.67–4.23) times and 3.01 (95% CI: 1.23–5.11) times higher of being MMFI compared with 2–3 family member counterparts, but the risk of being SFI was 2.91 (95% CI: 1.52–4.48) times higher among 4–5 family members in the household than 2–3 family member counterparts. The odds of being MMFI and SFI were 4.38 (95% CI: 1.33–7.61) times and 3.55 (95% CI: 2.38–4.69) times more likely in married respondent’s households compared with unmarried respondents counterparts. On the other hand, the results also showed that households who had moderate DDS and low DDS were 2.58 (95% CI: 1.14–4.38) times and 4.92 (95% CI: 1.87–7.61) times higher odds of being MMFI in comparison with peers with high DDS. Moreover, respondents with lower dietary diversity were 2.49 (95% CI: 0.94–2.94) times more likely to be severely food insecure than those with higher dietary diversity.

Effects on income and food accessibility were positively correlated with food insecurity across households during COVID-19 lockdown. The odds of being MMFI and SFI were 3.87 (95% CI: 1.37–6.46) times and 2.99 (95% CI: 1.16–4.83) times more likely in less income (not enough for food) respondents household compared with no changes in income counterparts. Households with changes in the type of cooked food were 2.62 times and 1.81 times more likely to be MMFI and SFI than households with no changes in the type of cooked food. In terms of reasons for the changes in the type of cooked food during COVID-19 lockdown, respondents with poor income and more household members were 3.43 times and 2.65 times higher odds of being MMFI than respondents who had no reason. In addition, household heads who had poor income were 2.70 (95% CI: 1.19–4.23) times more likely to be SFI relative to no reason peers (p<0.01). Moreover, respondents households who didn’t get the same amount of food and types of food as before COVID-19 lockdown were 3.21 times and 1.18 times higher of being MMFI; 3.40 times and 1.99 times higher of being SFI compared with households that get the same amount of food and types of food as before COVID-19 lockdown. Concerning the total household income, during the COVID-19 pandemic lockdown, households whose income didn’t remain the same were 3.41 (95% CI: 1.33–5.62) times and 2.60 (95% CI: 0.99–4.24) times more likely to experience MMFI, and SFI during the lockdown than households whose income remained the same period.

**Fig 2:**
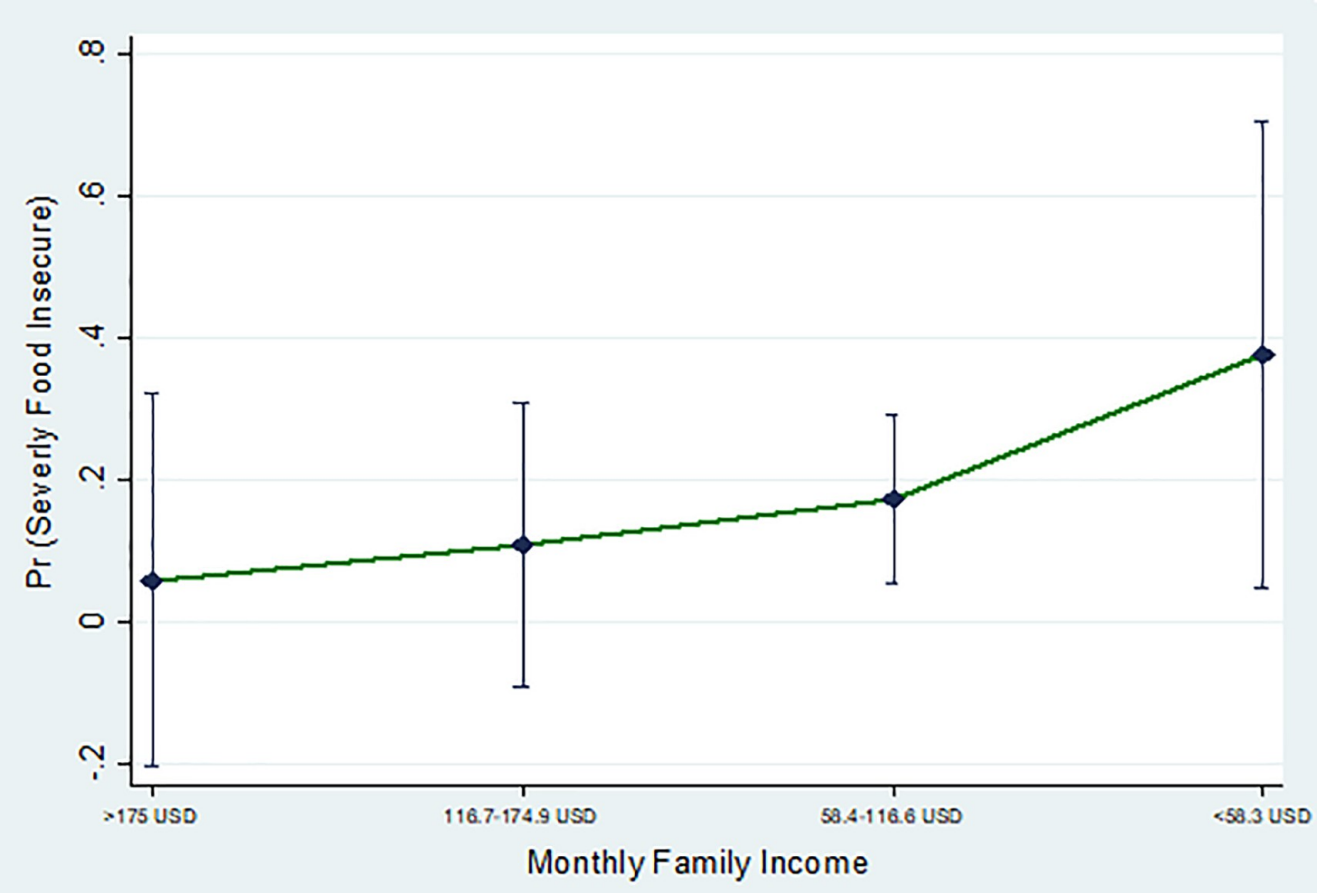
Predictive marginal effect of food insecurity with monthly family income.

**Fig 3:**
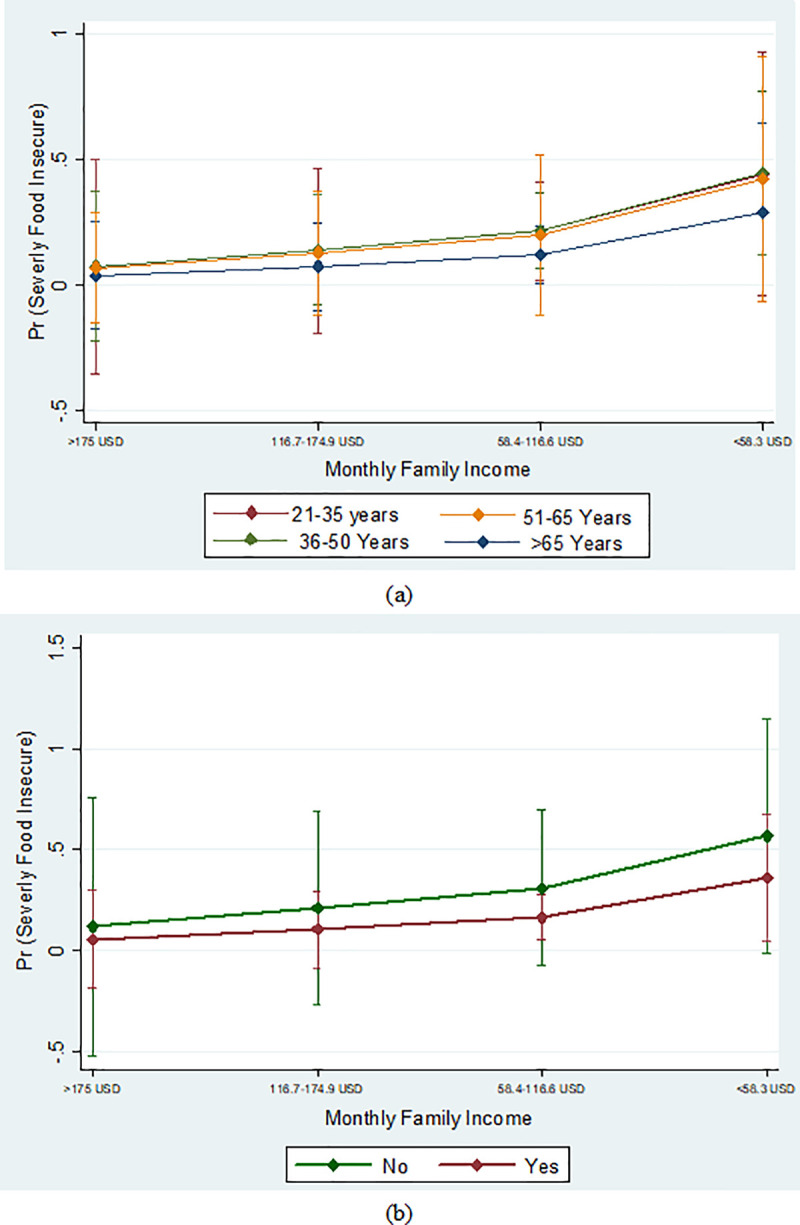
(a, b) Predictive marginal effect of food insecurity with monthly family income, age and income effect.

Fig 2 shows the predictive marginal effect of food insecurity with monthly family income with a 5% level of significance. Fig 2 shows that respondents who had monthly income below 58.3 USD and 58.4–116.6 USD were at comparatively higher risk of severe food insecurity than respondents’ monthly income above 175 USD. Fig 3A and 3B describes the predicted marginal effect of food insecurity with monthly family income, age, and earned the same type of income during the COVID-19 lockdown. These plots compared the probability of food insecurity of respondents who were in different monthly family income, ages, and earned the same type of income during the COVID-19 lockdown. The figure explains that respondents who were 36–50 years, 21–35 years had a higher risk of severely food insecure than >65 years respondents (Fig 3A). This figure also illuminates that both monthly family income and age were important factors for increasing the risk of food insecurity (Fig 3A). Above this, it also explains that people who were 36–50 years and 21–35 years old (monthly income below 58.3 USD and 58.4–116.6 USD) had more risk of being food insecure than >65 years respondents. Fig 3B shows that people who didn’t earn the same type of income as before during the COVID-19 lockdown had a higher risk of being food insecure than those who earned the same type of income as before. This figure also explained that both earned the same type of income during the COVID-19 lockdown and monthly family income was a significant factor to increase the risk of food insecurity.

## 4. Discussion

The emergence of the COVID-19 pandemic has not only affected the health of billions of people around the world but has also introduced food insecurity to their households. In response to global concerns on the impact of COVID-19 on food and nutrition security, the present study showed the prevalence of household food insecurity and associated factors during the COVID-19 lockdown among Bangladeshi lower-income households. The finding of this study indicated that more than 90 percent of the households experienced a different form of food insecurity (mild-to-moderate and severe food insecurity) during the lockdown period, while only 9.4 percent experienced food security. The findings are similar to the other recent studies in Bangladesh [8, 9]. Some recent evidence is available to support our findings that the prevalence of food insecurity is increased during the COVID-19 pandemic lockdown [21, 25, 32, 33]. The unprecedented COVID-19 pandemic, and the associated social and economic response have the potential to dramatically increase food insecurity and its related health disparities among already at-risk populations [34]. This study also explores the associated factors that significantly influenced household food insecurity are food accessibility and socioeconomic factors during the COVID-19 pandemic lockdown [25]. Various studies found some associated factors that significantly triggered food insecurity are age, education, household size, income, etc. during the pandemic [35–37].

This regression analysis indicated that different socioeconomic factors were positively associated with food insecurity during the COVID-19 pandemic lockdown. In this finding, young adults (21–35 years) and middle-aged (36–50 years) household heads were highly food insecure than older-aged counterparts. It is also evident that during the COVID-19 lockdown most of the low-income middle-aged and young adult household heads are losing their employment and income, making this a double crisis of food insecurity [1, 25, 38]. The result of this study showed that day labor and rickshaw puller respondents were more likely to be mild-to-moderately food insecure (MMFI) and severely food insecure (SFI) during the lockdown. Importantly, poor household income was significantly associated with household food insecurity during the COVID-19 pandemic. In this study, respondents with monthly income <58.3 USD and 58.4–116.6 USD were more likely to be food insecure. Recent population-based studies explained that poor household income significantly influenced the probability of food insecurity [18]. The prevalence of mild-to-moderate to severe food insecurity was higher among poor income households because of their low income, income reduction, or running out of savings during the COVID-19 lockdown [4, 39, 40]. Due to the fast spread of the COVID-19 pandemic, Bangladesh imposed the longest and strictest lockdowns which are particularly going to hit the low-income people (including day labor, rickshaw puller, hotel workers, and street vendors) with reduced employment opportunities, lower earnings, and disruptions to the supply chain threaten to worsen the food insecurity [9, 22, 24, 41].

This study also revealed that an increase of total household family members had a significantly higher probability of being SFI during the COVID-19 pandemic. Different global researchers also found similar findings [25, 42]. Besides, the low DDS status of the household also significantly influenced the likelihood of experiencing food insecurity. Two recent studies investigated that low dietary diversity scores mediate the effect of household foods insecurity [8, 25]. Previous research has also revealed that household food security influences the consumption of diverse diets [42, 43]. In fact, some studies also argue that dietary diversity can be used as a proxy for household food security status [44, 45]. Due to financial constraints, the households may purchase staples rather than a diverse diet. The positive correlation between the two could be explained by poverty, poor income, household size, and higher prices, which has been proven to be a strong predictor of both access to enough and diversified food [25, 42, 46].

Effects on income conditions and food accessibility are severely affected in low-income household heads during COVID-19 lockdown time, which is also associated with food insecurity. This study results reported that respondents with poor income (not enough for food purchase) had a higher probability of being MMFI. Importantly, this study also found that an increase in food prices during the COVID-19 pandemic lockdown significantly influenced the likelihood of households experiencing food insecurity. In addition, respondents who didn’t get the same quantity and quality of food as before the COVID-19 pandemic lockdown were more likely to be SFI. Moreover, above 92% of respondents didn’t earn the same amount of income as before the COVID-19 pandemic lockdown were significantly associated with food insecurity. Some community-based studies from different countries evaluated that food insecurity is strongly associated with daily poor income or loss of income on daily labor [18, 22, 38, 39]. Besides, recent Bangladeshi studies investigated that more than 70% of the daily wage earners had decreased or low income during the COVID-19 lockdown, and their households fall relatively more into both mild/moderate to the severe category of food insecurity [9, 24, 41]. Some potential explanation could be that imposed of countrywide strict lockdown most of the poor income people are being unemployed. Moreover, open-air food markets and small food shops are closed which creates more complexities for the daily wage-earning people [18]. Besides, the food supply chain was also disturbed due to public transport restrictions. For this reason, low-income people become more vulnerable to food insecurity as a consequence of a sudden drop in income, supply, or access to food [1, 8, 41]. With the COVID-19 pandemic lockdown, higher retail prices, reduced incomes, and poor availability may have reduced their purchase ability of preferred types and quality of foods as well as determining risk for food insecurity [8, 23, 24, 38]. Finally, our study results confirm that income loss, closure of public transport and open-air food markets, higher retail prices, and poor resources might be associated with moderate to severe food insecurity among low-income people.

Since the COVID-19 outbreak has rapidly spread all over the world, the subsequent lockdown has raised the risk of food and nutrition insecurity which poses a significant global public health threat. In developing countries like Bangladesh, food insecurity is rising during the pandemic. Our study results also explore that socioeconomic, income, and food access-related factors were strongly associated with households’ mild/moderate food insecurity and severe food insecurity among low-income people during the COVID-19 pandemic lockdown period. Knowledge regarding associated risk factors of households’ food insecurity is important to government and policymakers to initiate proper actions to lessen the prevalence of households’ food insecurity among low-income people. The study findings confirm the need for effective interventions to reduce the prevalence of households’ food insecurity among low-income people households during the COVID-19 pandemic. Our survey evidence suggests that government and policymakers should be designed appropriate policy and support to identify the low-income households during this pandemic lockdown period. Important policy actions including economic and financial support via direct payments or free food packages from the government might be a potential role to improve food insecurity status. Besides, the government should also consider collaborating with NGOs to provide loans for small and medium-sized enterprises which can boost people’s capability to tackle vulnerability to households’ food insecurity during the COVID-19 pandemic. In line with the COVID-19 response, providing psychological and behavioral programs on TV and social media can be recommended to lessen family stresses during the lockdown period.

### 4.1 Strength and limitation

The present study has some major strengths. Firstly, perhaps this is the first survey that evaluates the impact of COVID-19 pandemic lockdown on households’ food insecurity in Bangladeshi low-income people. Secondly, the study used a face-to-face survey to identify respondent households that integrated a large number of socioeconomic, income, and food access characteristics as confounders that are associated with food insecurity. In contrast, the object sample size was achieved during the pandemic, indicated the strength of this study, because we had significant power to test our hypotheses. Finally, this study also presents a nationally representative sample from three divisions to offer precise estimates on the association. This study has not beyond some limitations. First, the cross-sectional study design precludes any causal inferences between the outcome (food insecurity) and the predictors. Second, this research didn’t include minority household groups. Third, the dietary diversity score is calculated solely on 24-hour food consumption and hence could be subject to typical day-to-day variability, and it also does not account for the amount of food consumed. Fourth, causation cannot be established due to the cross-sectional design. Moreover, a significant limitation of this study is that all the information was self-reported and was based on subjective perceptions.

## 5. Conclusion

Countrywide strict lockdown imposed to protect the spread of COVID-19 pandemic which triggered a negative impact on income loss and food insecurity across household’s low-income people in Bangladesh. According to the findings of this study, low-income households experienced mild-to-moderate and severe food insecurity during the lockdown period. The risk of food insecurity was strongly associated with various factors as socioeconomic characteristics, dietary diversity score, the effect of income, and food prices. This study also showed that a small proportion of households received food from the government and other relief assistance during the COVID-19 lockdown. Therefore government and other organizations should step up to assist selected low-income households in reducing household food insecurity during the COVID-19 lockdown.

## Supporting information

S1 Fig (DOCX)

S1 Appendix Questionnaire. (DOCX)

S1 File Ad-hoc statistical analysis. (DOCX)

## Abbreviations

FI: Food insecurity
MMFI: Mild to moderate food insecure
SFI: Severely food insecure
DDS: Dietary diversity scores
FS: Food security
HFIAS: Household Food Insecurity Access Scale
