# Supplementary material for: Factors influencing low-income households’ food insecurity in Bangladesh during the COVID-19 lockdown
Source: PLoS One. 2022 May 10;17(5):e0267488. doi: 10.1371/journal.pone.0267488 (PMC9089875; doi:10.1371/journal.pone.0267488)
Supplement: S1 Fig — (DOCX) [file pone.0267488.s001.docx]

**Factors influencing low-income households’ food insecurity in Bangladesh during the COVID-19 lockdown**

|  |
| --- |
| (a) |
|  |
| (b) |
|  |
| (c) |

**Figure 4 (a, b, c):** Predictive marginal effect of Food Insecurity with monthly family income, age and earned the same type of income during the COVID-19 lockdown
