# Supplementary material for: Factors influencing low-income households’ food insecurity in Bangladesh during the COVID-19 lockdown
Source: PLoS One. 2022 May 10;17(5):e0267488. doi: 10.1371/journal.pone.0267488 (PMC9089875; doi:10.1371/journal.pone.0267488)
Supplement: S1 Appendix — (DOCX) [file pone.0267488.s002.docx]

**Factors influencing low-income households’ food insecurity in Bangladesh during the COVID-19 lockdown**

1. **Socio-demographic status of the respondents**

| **Name** |  | | | |
| --- | --- | --- | --- | --- |
| **Village/Town** |  | **Upazilla** |  | **District** |
| **Gender** | (0) Female | (1) Male |  |  |
| **Age** | (0) 21-35y | (1) 36-50y | (2) 51-65y | (3) >65y |
| **Level of education** | (0) Higher Secondary | (1) Secondary | (2) Primary | (3) Illiterate |
| **Occupation** | (1) Rikshawpullar | (2) Hotel Worker | (3) Day labor | (0) Other |
| **Family income (monthly)** | (0) >175USD | (1) 116.7-174.9 USD | (2) 58.4-116.6 USD | (3) <58.3 USD |
| **Family member** | (0) 2-3 | (1) 4-5 | (2) 6-7 | (3) ≥8 |
| **Marital Status** | (0) Unmaried | (1) Maried | (2) Widowed |  |

1. **Food access** **during COVID-19 lockdown**
2. **Effect on income during COVID-19 lockdown**

(0) No change

(1) Less income (not enough for food)

(2) Less income (but enough for food)

(3) No income coming into household

1. **Change in type of food cooked** **during COVID-19 lockdown**

(0) No

(1) Yes

1. **Change in cooking frequency during COVID-19 lockdown**

(0) No change

(1) More frequent

(2) Much less frequent

(3) Less frequent

1. **Reasons for change in type of food cooked** **during COVID-19 lockdown**

(0) More people in household

(1) Lower availability of cooking fuel

(2) Lower availability of food

(3) Lower income

1. **Food source during lockdown during COVID-19 lockdown**

(0) Local shop/market (same as before lockdown)

(1) Local shop/market (different location than before lockdown)

(2) Source from Govt. Relief /Help assistant

(3) Friends/family/source from home (different than before lockdown)

1. **Increase of food prices due to COVID-19 lockdown**

(0) No

(1) Don’t know

(2) Yes

1. **Get same amount of food as before COVID-19 lockdown**

(0) No

(1) Yes

1. **Get same type of food as before COVID-19 lockdown**

(0) No

(1) Yes

1. **Get same type of income as before COVID-19 lockdown----**

(0) No

(1) Yes

1. **Household Food Insecurity** **due to COVID-19 lockdown**

| **SN** | **Questionnaires** | **Response option** | **code** |
| --- | --- | --- | --- |
| **1.** | In the past four weeks, **did you worry** that your household would not have enough food due to COVID-19 lockdown?  **1.a How often did this happen?**  1 = Rarely (once or twice in the past four weeks)  2 = Sometimes (three to ten times in the past four weeks)  3 = Often (more than ten times in the past four weeks) | 0=No (skip to Q2)  1=Yes |  |
| **2.** | In the past four weeks, were **you or any household member not able to eat the kinds of foods** you preferred because of a lack of resources due to COVID-19 lockdown?  **2. a How often did this happen?**  1 = Rarely (once or twice in the past four weeks)  2 = Sometimes (three to ten times in the past four weeks)  3 = Often (more than ten times in the past four weeks) | 0=No (skip to Q3)  1=Yes |  |
| **3.** | In the past four weeks, **did you or any household member have to eat a limited variety of foods** due to a lack of resources due to COVID-19 lockdown?  **3. a How often did this happen?**  1 = Rarely (once or twice in the past four weeks)  2 = Sometimes (three to ten times in the past four weeks)  3 = Often (more than ten times in the past four weeks) | 0=No (skip to Q4)  1=Yes |  |
| **4.** | In the past four weeks, **did you or any household member have to eat some foods that you really did not want to eat** because of a lack of resources to obtain other types of food due to COVID-19 lockdown?  **4. a How often did this happen?**  1 = Rarely (once or twice in the past four weeks)  2 = Sometimes (three to ten times in the past four weeks)  3 = Often (more than ten times in the past four weeks) | 0=No (skip to Q5)  1=Yes |  |
| **5.** | In the past four weeks, **did you or any household member have to eat a smaller meal than you felt you needed** because there was not enough food due to COVID-19 lockdown?  **5. a How often did this happen?**  1 = Rarely (once or twice in the past four weeks)  2 = Sometimes (three to ten times in the past four weeks)  3 = Often (more than ten times in the past four weeks) | 0=No(skip to Q6)  1=Yes |  |
| **6.** | In the past four weeks, **did you or any other household member have to eat fewer meals in a day** because there was not enough food due to COVID-19 lockdown?  **6. a How often did this happen?**  1 = Rarely (once or twice in the past four weeks)  2 = Sometimes (three to ten times in the past four weeks)  3 = Often (more than ten times in the past four week) | 0=No (skip to Q7)  1=Yes |  |
| **7.** | In the past four weeks, **was there ever no food to eat of any time in your household member** because of lack of resources due to COVID-19 lockdown?  **7. a How often did this happen?**  1 = Rarely (once or twice in the past four weeks)  2 = Sometimes (three to ten times in the past four weeks)  3 = Often (more than ten times in the past four weeks) | 0=No (skip to Q8)  1=Yes |  |
| **8.** | In the past four weeks, **did you or any household member go to sleep at night hungry** because there was not enough food due to COVID-19 lockdown?  **8 .a How often did this happen?**  1 = Rarely (once or twice in the past four weeks)  2 = Sometimes (three to ten times in the past four weeks)  3 = Often (more than ten times in the past four weeks) | 0=No (skip to Q9)  1=Yes |  |
| **9.** | In the past four weeks, **did you or any household member go a whole day and night without eating anything** because there was not enough food due to COVID-19 lockdown?  **9. a How often did this happen?**  1 = Rarely (once or twice in the past four weeks)  2 = Sometimes (three to ten times in the past four weeks)  3 = Often (more than ten times in the past four week) | 0=No  1=Yes |  |

1. **Past 24 hour Dietary Diversity Score during COVID-19 lockdown**

| **Food groups included in dietary diversity score** | **Point** |
| --- | --- |
| 1. Any bread, rice, noodles, biscuits, or any other foods made from millet, sorghum, maize, rice, wheat or any other locally available grain | 1 |
| 1. Any potatoes, yams, manioc, cassava or any other foods made from roots or tubers | 1 |
| 1. Any Vegetables | 1 |
| 1. Any Fruits | 1 |
| 1. Any Meat, poultry, and other birds, liver kidney, heart or other organ meats | 1 |
| 1. Any fresh, dried fish or shellfish | 1 |
| 1. Any Eggs | 1 |
| 1. Any foods made from beans, peas, lentils or nuts | 1 |
| 1. Any cheese, yoghurt, milk or other milk products | 1 |
| 1. Any foods made with oil, fat or butter | 1 |
| 1. Any sugar or honey | 1 |
| 1. Any other foods such as condiments, coffee or tea | 1 |
| **Total** | **12** |

*Key: If the answer is “YES” then award 1 point: If the answer is “No” award 0 points*
