# Supplementary material for: Factors influencing low-income households’ food insecurity in Bangladesh during the COVID-19 lockdown
Source: PLoS One. 2022 May 10;17(5):e0267488. doi: 10.1371/journal.pone.0267488 (PMC9089875; doi:10.1371/journal.pone.0267488)
Supplement: S1 File — (DOCX) [file pone.0267488.s003.docx]

**Factors influencing low-income households’ food insecurity in Bangladesh during the COVID-19 lockdown**

**Table 1: Multivariate logit regression model results for the determinants of household food insecurity**

| **Variables** | **Mild-to-moderately food insecurity** | **Severe food insecurity** |  |
| --- | --- | --- | --- |
|  | ***RRR (95%CI)*** | ***RRR (95%CI)*** |  |
| **Family income per month (reference category:** **above 175 USD)** | | |  |
| <58.3 USD | 3.12 (1.16-5.18)*** | 3.34 (1.83-4.75)*** |  |
| 58.4-116.6 USD | 4.50 (1.56-7.85)** | 2.83 (0.90-4.82)** |  |
| 116.7-174.9 USD | 1.90 (0.50-3.31) | 1.56 (0.25-1.87) |  |
| **Age (reference category:** **>65 years)** | | |  |
| 21-35 years | 3.95 (1.94-5.88)** | 2.40 (0.88-3.81)** |  |
| 36-50 years | 4.78 (2.26-7.41)*** | 4.08 (2.20-6.07)*** |  |
| 51-65 years | 1.60 (0.46-2.64) | 3.07 (0.80-5.29) |  |
| **Level of education (reference category: higher secondary)** | |  |  |
| Illiterate | 2.86 (1.13-5.57)*** | 2.52 (1.18-3.80)*** |  |
| Primary | 2.10 (1.21-4.43)** | 2.38 (1.20-4.64)** |  |
| Secondary | 3.61 (2.43-5.25)** | 2.81 (1.34-4.40) ** |  |
| **Occupation (reference category: others)** | | |  |
| Day laborer | 3.20 (1.80-4.56) ** | 2.74 (1.20-4.22) |  |
| Rickshaw puller | 4.44 (1.90-7.34)** | 3.84 (1.45-6.43)** |  |
| Hotel Worker | 2.63 (1.40-4.39) | 1.96 (0.25-3.63) |  |
| **Marital Status (reference category: Unmarried)** | | |  |
| Married | 4.30 (1.30-7.56) *** | 3.45 (2.30-4.67)*** |  |
| Widowed | 1.15 (0.45-1.80) | 2.00 (0.86-3.21) |  |
| Observation | 500 |  |  |
| Log likelihood | -356.29 |  |  |
| *P*-value | <0.001 |  |  |
| LR chi^2^ (*χ^2^*) | 114.54 |  |  |
| Pseudo R^2^ (*ρ^2^*) | 0.13 |  |  |

**Table 2: Multivariate logit regression model results for the determinants of household food insecurity**

| **Variables** | **Mild-to-moderately food insecurity** | **Severe food insecurity** |
| --- | --- | --- |
|  | ***RRR (95%CI)*** | ***RRR (95%CI)*** |
| **Family income per month (reference category:** **above 175 USD)** | |  |
| <58.3 USD | 3.12 (1.16-5.18)*** | 3.34 (1.83-4.75)*** |
| 58.4-116.6 USD | 4.50 (1.56-7.85)** | 2.83 (0.90-4.82)** |
| 116.7-174.9 USD | 1.90 (0.50-3.31) | 1.56 (0.25-1.87) |
| 21-35 years | 3.95 (1.94-5.88)** | 2.40 (0.88-3.81)** |
| 36-50 years | 4.78 (2.26-7.41)*** | 4.08 (2.20-6.07)*** |
| 51-65 years | 1.60 (0.46-2.64) | 3.07 (0.80-5.29) |
| **Level of education (reference category: higher secondary)** | | |
| Illiterate | 2.86 (1.13-5.57)*** | 2.52 (1.18-3.80)*** |
| Primary | 2.10 (1.21-4.43)** | 2.38 (1.20-4.64)** |
| Secondary | 3.61 (2.43-5.25)** | 2.81 (1.34-4.40) ** |
| Day laborer | 3.20 (1.80-4.56) ** | 2.74 (1.20-4.22) |
| Rickshaw puller | 4.44 (1.90-7.34)** | 3.84 (1.45-6.43)** |
| Hotel Worker | 2.63 (1.40-4.39) | 1.96 (0.25-3.63) |
| **Marital Status (reference category: Unmarried)** | |  |
| Married | 4.30 (1.30-7.56) *** | 3.45 (2.30-4.67)*** |
| Widowed | 1.15 (0.45-1.80) | 2.00 (0.86-3.21) |
| **Family member (reference category:** 2-3**)** | |  |
| 4-5 | 2.45 (0.70- 4.28)** | 2. 99 (1.55-4.53)** |
| 6-7 | 2.04 (0.74-3.47) | 2. 09 (0.74-3.53) |
| ≥8 | 3.09 (1.25-5.16)** | 1.43 (0.35-2.49)** |
| **DDS (reference category:** **Good DDS)** |  |  |
| Moderate DDS | 2.70 (1.14-4.38) | 0.73 (0.20-1.19) |
| Low DDS | 4.81(1.86-4.75)** | 2.75(1.12-4.47)** |
| **Effect on income (reference category: No change)** | | |
| Less income (not enough for food) | 3.95 (1.40-6.51)*** | 2.91 (1.08-4.79)*** |
| Less income (but enough for food) | 1.70 (0.17-2.21) | 1.50 (0.50-2.43) |
| No income coming into household | 2.59 (1.09-4.39) | 1.59 (0.10-3.24) |
| Observation | 500 |  |
| Log likelihood | -332.66 |  |
| *P*-value | <0.001 |  |
| LR chi^2^ (*χ^2^*) | 161.80 |  |
| Pseudo R^2^ (*ρ^2^*) | 0.19 |  |

**Table 3: Multivariate logit regression model results for the determinants of household food insecurity**

| **Variables** | **Mild-to-moderately food insecurity** | **Severe food insecurity** |
| --- | --- | --- |
|  | ***RRR (95%CI)*** | ***RRR (95%CI)*** |
| **Family income per month (reference category:** **above 175 USD)** | |  |
| <58.3 USD | 3.12 (1.16-5.18)*** | 3.34 (1.83-4.75)*** |
| 58.4-116.6 USD | 4.50 (1.56-7.85)** | 2.83 (0.90-4.82)** |
| 116.7-174.9 USD | 1.90 (0.50-3.31) | 1.56 (0.25-1.87) |
| **Age (reference category:** **>65 years)** |  |  |
| 21-35 years | 3.95 (1.94-5.88)** | 2.40 (0.88-3.81)** |
| 36-50 years | 4.78 (2.26-7.41)*** | 4.08 (2.20-6.07)*** |
| 51-65 years | 1.60 (0.46-2.64) | 3.07 (0.80-5.29) |
| **Level of education (reference category: higher secondary)** | | |
| Illiterate | 2.86 (1.13-5.57)*** | 2.52 (1.18-3.80)*** |
| Primary | 2.10 (1.21-4.43)** | 2.38 (1.20-4.64)** |
| Secondary | 3.61 (2.43-5.25)** | 2.81 (1.34-4.40) ** |
| **Occupation (reference category: others)** |  |  |
| Day laborer | **3.20 (1.80-4.56) **** | 2.74 (1.20-4.22) |
| Rickshaw puller | 4.44 (1.90-7.34)** | 3.84 (1.45-6.43)** |
| Hotel Worker | 2.63 (1.40-4.39) | 1.96 (0.25-3.63) |
| **Marital Status (reference category: Unmarried)** | | |
| Married | 4.30 (1.30-7.56) *** | 3.45 (2.30-4.67)*** |
| Widowed | 1.15 (0.45-1.80) | 2.00 (0.86-3.21) |
| **Change in type of cooked** **food (reference category:** **No)** | | |
| Yes | 2.70 (1.40-3.82) | 1 .89 (0.25-3.46) |
| **Reasons for change in the type of cooked** **food (reference category:** **No reason)** | | |
| More people in household | 2.73 (1.16-4.17)** | 2.40(0.88-2.91)** |
| Lower availability of food | 1.21 (0.50-2.08)** | 1.44 (0.04-3.03) |
| Poor income | 3.51 (1.52-5.50)*** | 2.78 (1.25-4.25)*** |
| **Increase in food prices (reference category:** **No)** | | |
| Don’t know | 0.91 (0.01-1.96) | 0.28 (0.08-0.42) |
| Yes | 1.21 (0.28-2.27) | 1.16 (0.09-1.16) |
| Observation | 500 |  |
| Log likelihood | -348.68 |  |
| *P*-value | <0.001 |  |
| LR chi^2^ (*χ^2^*) | 129.76 |  |
| Pseudo R^2^ (*ρ^2^*) | 0.16 |  |

**Table 4: Multivariate logit regression model results for the determinants of household food insecurity**

| **Variables** | **Mild-to-moderately food insecurity** | **Severe food insecurity** |  |
| --- | --- | --- | --- |
|  | ***RRR (95%CI)*** | ***RRR (95%CI)*** |  |
| **Family income per month (reference category:** **above 175 USD)** | |  |  |
| <58.3 USD | 3.12 (1.16-5.18)*** | 3.34 (1.83-4.75)*** |  |
| 58.4-116.6 USD | 4.50 (1.56-7.85)** | 2.83 (0.90-4.82)** |  |
| 116.7-174.9 USD | 1.90 (0.50-3.31) | 1.56 (0.25-1.87) |  |
| **Age (reference category:** **>65 years)** | | |  |
| 21-35 years | 3.95 (1.94-5.88)** | 2.40 (0.88-3.81)** |  |
| 36-50 years | 4.78 (2.26-7.41)*** | 4.08 (2.20-6.07)*** |  |
| 51-65 years | 1.60 (0.46-2.64) | 3.07 (0.80-5.29) |  |
| **Level of education (reference category: higher secondary)** | |  |  |
| Illiterate | 2.86 (1.13-5.57)*** | 2.52 (1.18-3.80)*** |  |
| Primary | 2.10 (1.21-4.43)** | 2.38 (1.20-4.64)** |  |
| Secondary | 3.61 (2.43-5.25)** | 2.81 (1.34-4.40) ** |  |
| **Occupation (reference category: others)** | | |  |
| Day laborer | 3.20 (1.80-4.56) ** | 2.74 (1.20-4.22) |  |
| Rickshaw puller | 4.44 (1.90-7.34)** | 3.84 (1.45-6.43)** |  |
| Hotel Worker | 2.63 (1.40-4.39) | 1.96 (0.25-3.63) |  |
| **Marital Status (reference category: Unmarried)** | | |  |
| Married | 4.30 (1.30-7.56) *** | 3.45 (2.30-4.67)*** |  |
| Widowed | 1.15 (0.45-1.80) | 2.00 (0.86-3.21) |  |
| **Get the same amount quantity of food as before (reference category: Yes)** | | |  |
| No | 3.29 (1.50-5.09)** | 3.48 (1.55-5.44)** |  |
| **Get the same quality of food as before (reference category: Yes)** | | |  |
| No | 1.26 (0.50-1.99) | 1.91 (0.45-3.46) |  |
| **Earned the same type of income as before (reference category:** **Yes)** | | |  |
| No | 3.49 (1.40-5.61)*** | 2.52 (0.95-4.20)*** |  |
| Observation | 500 |  |  |
| Log likelihood | -329.88 |  |  |
| *P*-value | <0.001 |  |  |
| LR chi^2^ (*χ^2^*) | 167.36 |  |  |
| Pseudo R^2^ (*ρ^2^*) | 0.21 |  |  |
